# Crystal structure of the two-dimensional coordination polymer poly[di-μ-bromido-bis­(μ-tetra­hydro­thiophene)­dicopper(I)]

**DOI:** 10.1107/S2056989021006460

**Published:** 2021-06-25

**Authors:** Michael Knorr, Lydie Viau, Yoann Rousselin, Marek M. Kubicki

**Affiliations:** a Institut UTINAM UMR CNRS 6213, Université Bourgogne Franche-Comté, 16 route de Gray, 25030 Besançon, France; b ICMUB UMR CNRS 6302, Université Bourgogne Franche-Comté, 9 avenue Alain Savary, 21078 Dijon, France

**Keywords:** crystal structure, two-dimensional coordination polymer, Cu_2_Br_2_ rhomboids, tetra­hydro­thio­phene, C—H⋯Br hydrogen bonding.

## Abstract

The polymeric title compound, [Cu_2_Br_2_(C_4_H_8_S)_2_]_*n*_, **CP1**, represents an example of a two-dimensional coordination polymer resulting from reaction of CuBr with tetra­hydro­thio­phene (THT) in MeCN solution. The two-dimensional layers consist of two different types of rhomboid-shaped dinuclear Cu(μ_2_-Br)_2_Cu secondary building units (SBUs); one with a quite loose Cu⋯Cu separation of 3.3348 (10) Å and a second one with a much closer inter­metallic contact of 2.9044 (9) Å. These SBUs are inter­connected through bridging THT ligands, in which the S atom acts as a four-electron donor bridging each Cu(μ_2_-Br)_2_Cu unit in a μ_2_-bonding mode. In the crystal, the layers are linked by very weak C—H⋯·Br hydrogen bonds with H⋯Br distances of 2.95 Å, thus giving rise to a three-dimensional supra­molecular network.

## Abstract 2

Treatment of CuBr with tetra­hydro­thio­phene produces a layered coordination polymer.

## Chemical context   

The five-membered heterocyclic ligand tetra­hydro­thio­phene (THT) is known to form a great variety of mol­ecular complexes and coordination polymers (CPs) with various transition metals. In addition, numerous structurally characterized examples coordinated by terminal or bridging THT ligands have been documented for the soft coinage metal ions Cu^I^, Ag^I^ and Au^I^ (Usón *et al.*, 1984 ▸; Noren & Oskarsson, 1985 ▸; Mälger *et al.*, 1992 ▸; Ahrland *et al.*, 1993 ▸; López-De-Luzuriaga *et al.*, 1997 ▸; Ahrens & Jones, 2000 ▸). The research group of Pike has shown that depending on the reaction conditions, the treatment of CuI with THT affords dinuclear [(THT)_2_Cu(μ_2_-I)_2_Cu(THT)_2_], or the tetra­nuclear closed cubane-type cluster [(Cu_4_(μ_3_-I)_4_(THT)_4_] or [(CuI)_10_(THT)_7_(MeCN)]_*n*_ (Henline *et al.*, 2014 ▸). The latter contains the mixed motif [(Cu_4_I_4_(THT)](μ_2_-THT)_2_(Cu_2_I_2_)(μ_2_-THT)_2_[Cu_4_I_4_(THT)] held together side-by-side by two μ_2_-THT assembling ligands to form a 1D ladder structure. Furthermore, the two-dimensional CP [(CuI)_3_(THT)_3_·MeCN]_*n*_ featuring a sheet structure in which Cu_3_(THT) rings are linked in trigonal directions by rhomboid Cu_2_I_2_ dimers is literature-known (Henline *et al.*, 2014 ▸). The luminescent product [(CuI)_4_(THT)_2_]_*n*_ consisting of Cu_4_I_4_ cubane units knit into a 3D network by μ_2_-THT ligands was also described previously (Noren & Oskarsson, 1987 ▸; Henline *et al.*, 2014 ▸). A series of solvent-dependent 2D polymers results from treatment of [Cu(CO)Cl]_*n*_ with THT in THF, CH_2_Cl_2_ and DMF, exhibiting the composition [(CuCl)(THT)]_*n*_ (THF), [(CuCl)(THT)]_*n*_ (CH_2_Cl_2_), and [(CuCl)_3_(THT)_2_]_*n*_ (DMF), respectively. The materials obtained in THF and CH_2_Cl_2_ are polymorphs (Solari *et al.*, 1996 ▸). A mono-dimensional ribbon [(CuCl)_2_(THT)_3_]_*n*_ is generated by reaction of CuCl in neat THT (Mälger *et al.*, 1992 ▸). Even mixed-valence Cu^I^/Cu^II^ compounds such as polymeric penta-μ-chloro-tris-μ-tetra­hydro­thio­phene-tetra­copper(I,II) have been observed (Ainscough *et al.*, 1985 ▸). Mälger and co-workers also showed that the treatment of CuBr in neat THT leads to the formation of a very labile rhomboid-based 1D polymer of the type [(CuBr)_2_(THT)_3_]_*n*_ isostructural with its [(CuCl)_2_(THT)_3_]_*n*_ analogue (Mälger *et al.*, 1992 ▸) (CSD JUDKOI).
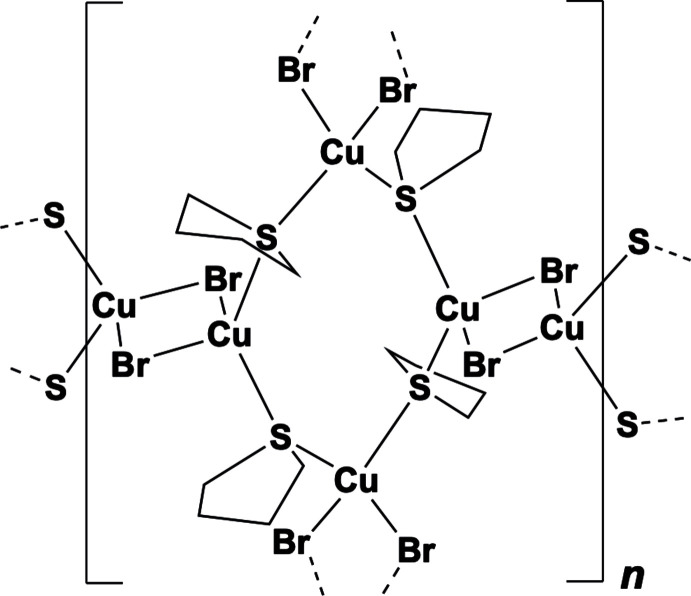


In the context of our research inter­est in the assembly of mol­ecular cluster compounds and coordination polymers by complexation of dialkyl sulfides *R*–*S*–*R* or di­thiol­ane- and di­thiane-based thia­heterocycles with Cu*X* salts (Knorr *et al.*, 2010 ▸, 2015 ▸; Lapprand *et al.*, 2013 ▸; Raghuvanshi *et al.*, 2017 ▸, 2019 ▸; Schlachter *et al.*, 2018 ▸; Knauer *et al.*, 2020 ▸), we have also investigated the complexation of CuBr by THT in aceto­nitrile as solvent (see Fig. 5 ▸) and present here the respective crystal structure, which differs both in composition and dimensionality (two-dimensional *vs* mono-dimensional) from the CP [(CuBr)_2_(THT)_3_]_*n*_ reported by Mälger. Note that this colourless material crystallizes easily in the form of large well-shaped crystals that are stable in a THT-saturated environment, but decomposes rapidly by dissociation of volatile THT upon exposure to air.

## Structural commentary   

**Figure 1:**
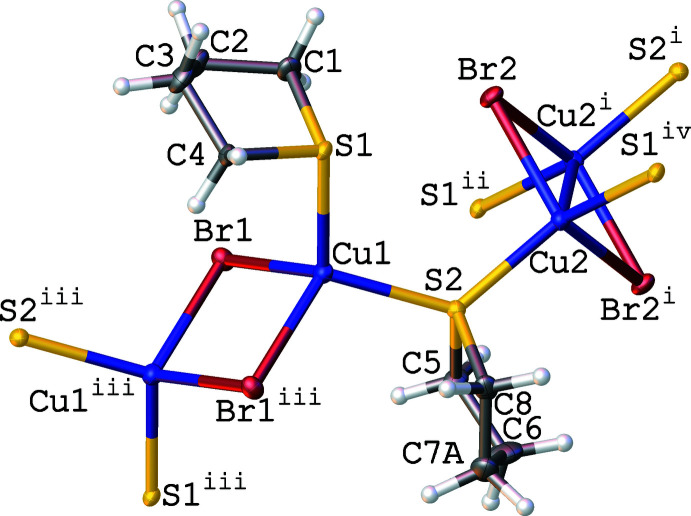
A view of **CP1** depicting the independent Cu_2_Br_2_ SBUs and THT ligands. Ellipsoids are shown at the 50% probability level. Only the major component of disordered atom C7 is shown. Symmetry codes: (i) −*x* + 2, −*y*, −*z* + 1; (ii) *x* + 1, *y*, *z*;) iii) −*x* + 1, −*y*, −*z*; (iv) −*x* + 1, −*y*, −*z* + 1.

**Figure 2:**
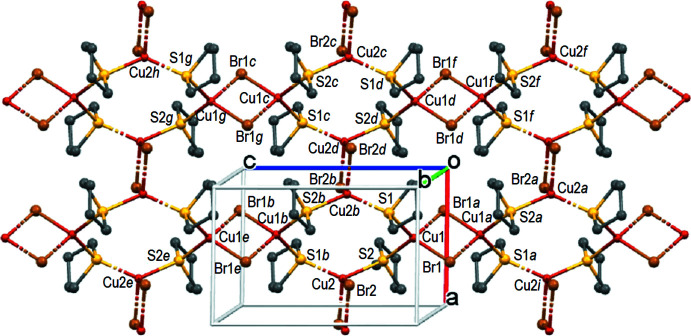
Projection of one layer on the ***a0c*** plane in the structure of **CP1**. Hydrogen atoms are omitted for clarity. Only the major component of disordered C7 atom is shown. Symmetry codes: (a) − *x* + 1, −*y*, −*z*; (b) −*x* + 1, −*y*, −*z* + 1; (c) −*x*, −*y*, −*z* + 1; (d) *x* − 1, *y*, *z*; (e) *x*, *y*, *z* + 1; (f) −*x*, −*y*, −*z*; (g) *x* − 1, *y*, *z* + 1; (h) −*x*, −*y*, −*z* + 2; (i) *x*, *y*, *z* + 1; (j) *x*, *y*, *z* − 1.

The crystal structure of **CP1** of composition [(CuBr)_2_(THT)_2_]_*n*_ is built of Cu(μ_2_-Br)_2_Cu rhomboids as SBUs and tetra­hydro­thio­phene ligands. The asymmetric unit contains two independent planar Cu_2_Br_2_ units placed over the symmetry centres at ½, 0, 0 (Cu1Br1)_2_ and 1, 0, ½ (Cu2Br2)_2_. They are connected through the sulfur atoms of thio­phene ligands acting, like in all bridging mono­thio­ethers, as four-electron donors (Fig. 1 ▸). The bridging S1 atoms develop the chains of alternating (Cu1Br1)_2_ and (Cu2Br2)_2_ SBUs parallel to one diagonal [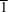
01] direction of the ***a0c*** face of the unit cell (labelled on Fig. 2 ▸ from Cu2*h* to Cu2*i*), whereas the S2 atoms develop the analogous chains labelled from Cu2*e* to Cu2*f* parallel to the second diagonal [101] direction of this face. The thus formed 2D layers lie over the (010) planes (Fig. 2 ▸). This is the essential difference from the 1D polymer [(CuBr)_2_(THT)_3_]_*n*_ described by Mälger (Mälger *et al.*, 1992 ▸) in which only one THT mol­ecule acts as a bridging ligand, developing a chain in one direction, whereas the two other THT mol­ecules are terminal. The outstanding feature of the structure of **CP1** consists of largely different (0.43 Å) Cu⋯Cu distances in (Cu1Br1)_2_ and (Cu2Br2)_2_ units [3.3348 (10) Å *vs* 2.9044 (9) Å], albeit in similar chemical surroundings. Contrary to these metal-to-metal separations, the Cu—Br and Cu—S bond lengths are similar in both rhomboids. In the 1D polymer of Mälger, the Cu⋯Cu distance of 2.7784 (7) Å is significantly shorter than in **CP1**. Note that the presence of two independent Cu_2_Br_2_ SBUs has been also reported for the structure of Cu_2_Br_2_(1,4-oxa­thiane)_2_ but the difference between the Cu⋯Cu distances therein is equal only to 0.12 Å [2.740 (3) Å *vs* 2.865 (4) Å; Barnes & Paton, 1982 ▸; CSD BOGTIA]. This difference is still smaller in two other CPs with different Cu_2_Br_2_ SBUs: [{Cu(μ_2_-Br)_2_Cu}{μ-PhS(CH_2_)_3_SPh}_2_]_*n*_ [*d*Cu⋯Cu = 2.794 (1) and 2.776 (1) Å; Knorr *et al.*, 2012 ▸; CSD ZEHREL] and in [{Cu(μ_2_-Br)_2_Cu}{μ-*p*-MeC_6_H_4_SCH_2_C≡CCH_2_SC_6_H_4_Me-*p*]_*n*_ [*d*Cu⋯Cu = 2.9306 (14) and 2.9662 (14) Å; Bonnot *et al.*, 2015 ▸; CSD QUPXOQ]. These observations indicate a high flexibility of the Cu_2_Br_2_ units. It is worth noting that the Cu⋯Cu distances in coordination polymers containing di­bromo­dicopper units and bridging mono­thio­ethers have been observed in the range from 2.740 (3) Å in Cu_2_Br_2_(1,4-oxa­thiane)_2_ (Barnes & Paton, 1982 ▸) to 3.074 (1) Å at 115 K in [(Cu_2_Br_2_)(Cu_4_Br_4_)(SMeEt)_6_]_*n*_ (Knorr *et al.*, 2010 ▸). Thus, the Cu1⋯ Cu1 distance of 3.3348 (10) Å in **CP1** is the longest one observed in Cu_2_Br_2_ CPs with bridging mono­thio­ethers. The coordination polyhedra of the Cu1 and Cu2 atoms are best described as distorted tetra­hedral. Even though the values of four-coord­inate geometry τ_4_ indexes of Yang (Yang *et al.*, 2007 ▸) of 0.88 support a trigonal–pyramidal geometry (theoretical values are equal to 0.85 for *C_3v_* and 1.0 for *T_d_* symmetries), the tetra­hedral character THC_DA_ parameters of Höpfl (0.66 for Cu1 and 0.60 for Cu2) are closer to the tetra­hedral (THC = 1.0) than pyramidal (THC = 0) geometries (Höpfl, 1999 ▸). Moreover, the sums of all six bond angles around Cu1 (656.7°) and Cu2 (656.1°) are very close to the value expected for *T_d_* symmetry (657°) and far from that of 630° in an ideal trigonal–pyramidal geometry.

## Supra­molecular features   

**Figure 3:**
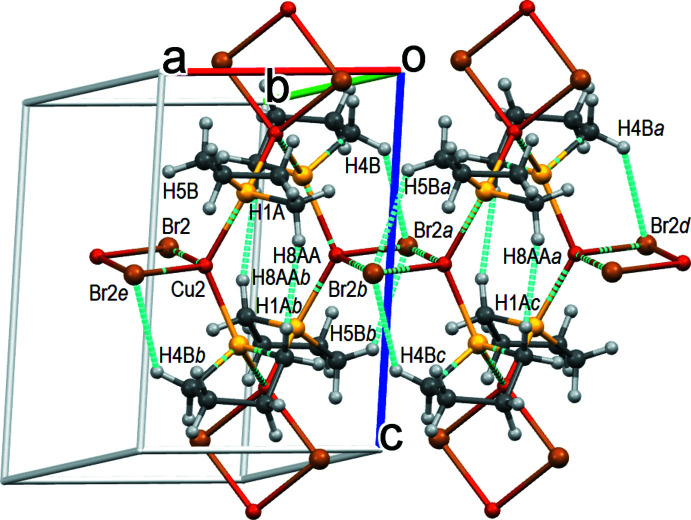
Intra­layer CH⋯Br and CH⋯HC non-covalent inter­actions. Symmetry codes: (a) *x* − 1, *y*, *z*; (b) −*x* + 1, −*y*, −*z* + 1; (c) −*x*, −*y*, −*z* + 1; (d) *x* − 2, *y*, *z*; (e) −*x* + 2, −*y*, −*z* + 1.

**Figure 4:**
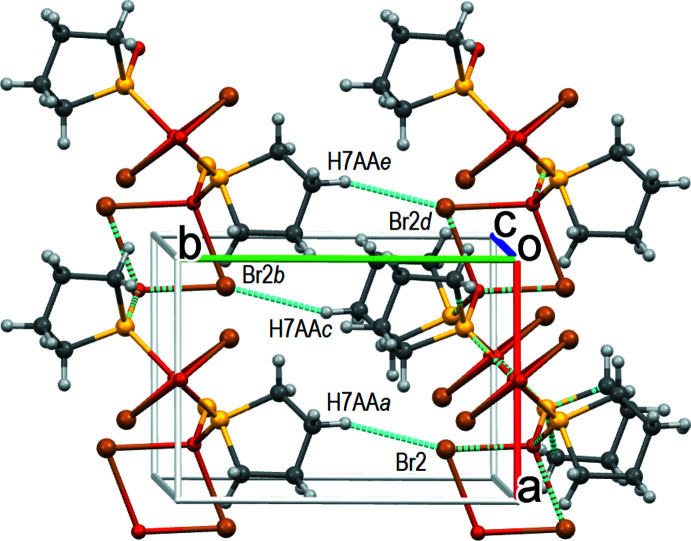
Inter­layer CH⋯Br hydrogen bonds. Symmetry codes: (a) *x*, *y* + 1, *z*; (b) −*x* + 1, −*y* + 1, −*z* + 1; (c) −*x* + 1, −*y*, −*z* + 1; (d) *x* − 1, *y*, *z*; *(*e) *x* − 1, *y* + 1, *z*.

The layers are built through dative Cu—S coordination bonds. There are also weak non-covalent CH⋯HC [*d*(H1*A*⋯H8*AA*) = 2.36 Å] van der Waals contacts and C—H⋯Br [*d*(Br2⋯H4*B*) = 2.90 Å; *d*(Br2⋯H5*B*) = 2.89 Å] hydrogen bonds within the layers (Fig. 3 ▸ and Table 1 ▸). More inter­estingly, the inter­layer connectivity for formation of a supra­molecular 3D structure is apparently limited only to very weak C—H⋯Br hydrogen bonds (Fig. 4 ▸). The Br2⋯H7*AA* distance of 2.95 Å is shorter by only 0.10 Å than the sum of the van der Waals radii (Bondi, 1964 ▸). It is noteworthy that only bromine Br2 participates in hydrogen-bonding contacts and not the bromine atom Br1. In the Cu1Br1 rhomboids, the Br⋯ Br distance is short, the Cu⋯Cu distance is long and there are no Br⋯H bonds, while in the Cu2Br2 rhomboids the opposite is observed with long Br⋯Br and short Cu⋯Cu distances and the presence of Br2⋯H inter­actions. However, we don’t believe that the presence or absence of weak hydrogen bonding alone may explain the large difference in the Cu⋯Cu distances.

## Database survey   

The rich structural diversity of THT-ligated mol­ecular and polymeric copper(I) halide compounds was already laid out extensively in the *Chemical context* section above. Further examples found in a database survey using *CONQUEST* (Bruno *et al.*, 2002 ▸) comprise, for example, the three-dimensional MOF [tris­(μ_2_-cyano)-tris­(μ_2_-THT)tricopper(I)]_*n*_ (CSD ITEZOX), which was isolated upon treatment of CuCN with THT (Dembo *et al.*, 2010 ▸). An example of a cationic dinuclear bi­pyridine-bridged complex is (μ-4,4′-bi­pyridine)­bis­(THT)tetra­kis­(tri­phenyl­phosphine)di-copperbis(tetra­fluoro­borate) (CSD MOJWOZ; Royzman *et al.*, 2014 ▸). A structurally characterized mol­ecular organometallic aryl complex [2,6-bis­(2,4,6-triiso­propyl­phen­yl)phen­yl](THT)copper(I) (CSD DOPMUR) is another relevant contribution in this context (Groysman & Holm, 2009 ▸). There is also the inter­esting case of the tetra­nuclear compound *cyclo*[tetra­kis­(μ_2_-mesityl­idene)bis­(THT-copper)dicopper(I)] featuring bridging aryl groups and terminal bound THT ligands (Meyer *et al.*, 1989 ▸). For selected examples of mol­ecular thio­ether-ligated complexes incorporating dinuclear Cu(μ_2_-Br)_2_Cu SBUs, see: [{Cu(μ_2_-Br)_2_Cu}{1-oxa-4,7-di­thia­cyclo­nona­ne}_2_] [Lucas *et al.*, 1997 ▸; CSD NONWOC, *d*Cu⋯Cu = 2.852 (2) Å]; [{Cu(μ_2_-Br)_2_Cu}{phenyl propargyl sulfide}_4_] [Kokoli *et al.*, 2013 ▸; CSD VEQXUM, *d*Cu⋯Cu = 3.0062 (7) Å]. For selected examples of mono-dimensional thio­ether-assembled CPs incorporating dinuclear Cu(μ_2_-Br)_2_Cu SBUs, see: [{Cu(μ_2_-Br)_2_Cu}{μ-PhSCH_2_SPh}_2_]_*n*_ [Knorr *et al.*, 2014 ▸; CSD FOWZIC, *d*Cu⋯Cu = 2.9192 (8) Å]; [{Cu(μ_2_-Br)_2_Cu}{μ-PhS(CH_2_)_3_SPh}_2_]_*n*_ [Knorr *et al.*, 2012 ▸; CSD ZEHREL, *d*Cu⋯Cu = 2.794 (1) and 2.776 (1) Å]; [Cu(μ_2_-Br)_2_Cu{μ-*p*-EtSCH_2_C_6_H_4_C_6_H_4_CH_2_SEt-*p*}_2_]_*n*_ [Toyota *et al.*, 1996 ▸; CSD ZARYUM01, *d*Cu⋯Cu = 2.918 (11) Å]; [Cu(μ_2_-Br)_2_Cu{μ-O_2_S_2_-macrocycle)_2_]_*n*_ [Park *et al.*, 2012 ▸; CSD GAXHIY, *d*Cu⋯Cu = 2.927 (1) Å].

For selected examples of two-dimensional thio­ether-assembled CPs incorporating dinuclear Cu(μ_2_-Br)_2_Cu SBUs, see: [{Cu_2_(μ_2_-Br)_2_}(tetra­thia­phthalazinophane)_2_]_*n*_ [Chen *et al.*, 1993 ▸; CSD HANGUY, *d*Cu⋯Cu = 3.06 (8) Å]; [{Cu(μ_2_-Br)_2_Cu}(μ_2_-2-isobutyl-1,3-di­thiane)_2_]_*n*_ [Raghuvanshi *et al.*, 2019 ▸; CSD JIZQOB, *d*Cu⋯Cu = 2.9057 (8) Å]; [{Cu(μ_2_-Br)_2_Cu}{μ-PhCH_2_S(CH_2_)_6_SCH_2_Ph}_2_]_*n*_ [Schlachter *et al.*, 2020 ▸; CSD IHIBUZ, *d*Cu⋯Cu = 2.953 (3) Å]; [{Cu(μ_2_-Br)_2_Cu}{μ-PhCH_2_S(CH_2_)_7_SCH_2_Ph}_2_]_*n*_ [Schlachter *et al.*, 2020 ▸; CSD IHICOU, *d*Cu⋯Cu = 2.7081 (4) Å]; [{Cu(μ_2_-Br)_2_Cu}(μ-1,2,4,5-tetra­methyl­mercapto­benzene)]_*n*_ [Suenaga *et al.*, 1997 ▸; CSD WIQMIS, *d*Cu⋯Cu = 3.1073 (12) Å]. An evaluation of these examples emphasizes that the Cu⋯Cu separations within the dinuclear Cu(μ_2_-Br)_2_Cu SBUs are quite variable.

## Synthesis and crystallization   

**Figure 5:**
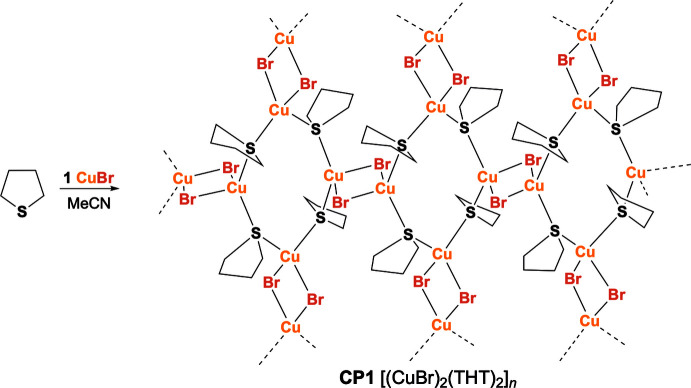
Reaction scheme for the synthesis of **CP1**.

To a solution of CuBr (1.43 g, 10.0 mmol) in MeCN (12 mL) was added neat THT (1.058 g, 12.0 mmol) *via* syringe. The solution turned brownish-red and a colourless microcrystalline material commenced to precipitate. The suspension was stirred at 293 K for 2 h, then heated 2 min to reflux until all product dissolved. While slowly warming to ambient temperature, colourless crystals formed progressively (Fig. 5 ▸). Filtering off the product after 1 d and storing the mother liquor in a refrigerator afforded a second crop of **CP1**. Overall yield (1.80 g, 78% yield). Calculated for C_8_H_16_Br_2_Cu_2_S_2_: C, 20.74 H, 3.48; S, 13.84. Found: C, 20.35; H, 3.28, S, 13.41%.

## Refinement   

Crystal data, data collection and structure refinement details are summarized in Table 2 ▸. All H atoms were placed in calculated positions and treated with a riding model. C—H distances were set to 0.99 Å with *U*_iso_(H) = *1.2U*_eq_(C). C7 in one of the THT ligands as well as the riding methyl­ene hydrogen atoms on C6, C7 and C8 are disordered over two locations. Their occupancy factors refined to 0.77 (1) and 0.23 (1). The disorder was modelled using a SADI constraint for the affected C—C distances.

## Supplementary Material

Crystal structure: contains datablock(s) I. DOI: 10.1107/S2056989021006460/yz2008sup1.cif

Structure factors: contains datablock(s) I. DOI: 10.1107/S2056989021006460/yz2008Isup2.hkl

CCDC reference: 2091214

Additional supporting information:  crystallographic information; 3D view; checkCIF report

## Figures and Tables

**Table 1:** Hydrogen-bond geometry (Å, °)

*D*—H⋯*A*	*D*—H	H⋯*A*	*D*⋯*A*	*D*—H⋯*A*
C4—H4*B*⋯Br2^iv^	0.99	2.90	3.566 (4)	125
C5—H5*B*⋯Br2^ii^	0.99	2.89	3.556 (4)	126
C7*A*—H7*AA*⋯Br2^v^	0.99	2.95	3.885 (6)	157

Symmetry codes: (ii) -x+2, -y, -z+1; (iv) x-1, y, z; (v) x, y-1, z.

**Table 2:** Experimental details

Crystal data	
Chemical formula	[Cu_2_Br_2_(C_4_H_8_S)_2_]
*M*_r_	463.23
Crystal system, space group	Triclinic, *P*\overline{1}
Temperature (K)	115
*a*, *b*, *c* (Å)	6.8076 (3), 9.7078 (4), 10.1579 (4)
α, β, γ (°)	75.804 (2), 89.845 (2), 89.594 (2)
*V* (Å^3^)	650.79 (5)
*Z*	2
Radiation type	Mo *K*α_1_
μ (mm^−1^)	9.69
Crystal size (mm)	0.25 × 0.15 × 0.1

Data collection	
Diffractometer	Nonius Kappa APEXII
Absorption correction	Multi-scan (Blessing, 1995 ▸)
*T*_min_, *T*_max_	0.024, 0.072
No. of measured, independent and observed [*I* > 2σ(*I*)] reflections	5297, 2954, 2743
*R*_int_	0.021
(sin θ/λ)_max_ (Å^−1^)	0.651

Refinement	
*R*[*F*^2^ > 2σ(*F* ^2^)], *wR*(*F* ^2^), *S*	0.030, 0.075, 1.09
No. of reflections	2954
No. of parameters	132
No. of restraints	6
H-atom treatment	H-atom parameters constrained
Δρ_max_, Δρ_min_ (e Å^−3^)	0.82, −0.91

Computer programs: *COLLECT* (Nonius, 1997 ▸), *HKL*, *DENZO* and *SCALEPACK* (Otwinowski & Minor 1997 ▸), *SIR97* (Burla *et al.*, 2007 ▸), *SHELXL2018/3* (Sheldrick, 2015 ▸) and *OLEX2* (Dolomanov *et al.*, 2009 ▸).

## supplementary crystallographic information

### Crystal data

**Table:**

[Cu_2_Br_2_(C_4_H_8_S)_2_]	*Z* = 2
*M**_r_* = 463.23	*F*(000) = 448
Triclinic, *P*1	*D*_x_ = 2.364 Mg m^−^^3^
*a* = 6.8076 (3) Å	Mo *K*α_1_ radiation, λ = 0.71073 Å
*b* = 9.7078 (4) Å	Cell parameters from 2662 reflections
*c* = 10.1579 (4) Å	θ = 1.0–27.5°
α = 75.804 (2)°	µ = 9.69 mm^−^^1^
β = 89.845 (2)°	*T* = 115 K
γ = 89.594 (2)°	Prism, clear light colourless
*V* = 650.79 (5) Å^3^	0.25 × 0.15 × 0.1 mm

### Data collection

**Table:**

Nonius Kappa APEXII diffractometer	2954 independent reflections
Radiation source: X-ray tube, Siemens KFF Mo 2K-180	2743 reflections with *I* > 2σ(*I*)
Graphite monochromator	*R*_int_ = 0.021
Detector resolution: 9 pixels mm^-1^	θ_max_ = 27.6°, θ_min_ = 3.0°
φ and ω scans'	*h* = −8→8
Absorption correction: multi-scan (Blessing, 1995)	*k* = −12→12
*T*_min_ = 0.024, *T*_max_ = 0.072	*l* = −12→13
5297 measured reflections	

### Refinement

**Table:**

Refinement on *F*^2^	Primary atom site location: structure-invariant direct methods
Least-squares matrix: full	Hydrogen site location: inferred from neighbouring sites
*R*[*F*^2^ > 2σ(*F*^2^)] = 0.030	H-atom parameters constrained
*wR*(*F*^2^) = 0.075	*w* = 1/[σ^2^(*F*_o_^2^) + (0.0333*P*)^2^ + 2.0511*P*] where *P* = (*F*_o_^2^ + 2*F*_c_^2^)/3
*S* = 1.09	(Δ/σ)_max_ < 0.001
2954 reflections	Δρ_max_ = 0.82 e Å^−^^3^
132 parameters	Δρ_min_ = −0.91 e Å^−^^3^
6 restraints	

### Special details

**Table:**

Geometry. All esds (except the esd in the dihedral angle between two l.s. planes) are estimated using the full covariance matrix. The cell esds are taken into account individually in the estimation of esds in distances, angles and torsion angles; correlations between esds in cell parameters are only used when they are defined by crystal symmetry. An approximate (isotropic) treatment of cell esds is used for estimating esds involving l.s. planes.

### Fractional atomic coordinates and isotropic or equivalent isotropic displacement parameters (Å^2^)

**Table:**

	*x*	*y*	*z*	*U*_iso_*/*U*_eq_	Occ. (<1)
Br1	0.66540 (5)	0.15468 (4)	−0.03837 (3)	0.01461 (10)	
Br2	0.83088 (5)	0.17939 (4)	0.45014 (4)	0.01506 (10)	
Cu1	0.51313 (7)	−0.00292 (5)	0.16455 (5)	0.01505 (11)	
Cu2	0.82082 (6)	−0.08288 (5)	0.51958 (4)	0.01385 (11)	
S1	0.31065 (12)	0.13645 (9)	0.26538 (8)	0.01195 (17)	
S2	0.70032 (12)	−0.14665 (9)	0.33197 (9)	0.01222 (17)	
C1	0.4015 (6)	0.3198 (4)	0.2177 (4)	0.0192 (8)	
H1A	0.375096	0.368406	0.290963	0.023*	
H1B	0.544740	0.320766	0.200413	0.023*	
C2	0.2910 (6)	0.3928 (4)	0.0890 (4)	0.0206 (8)	
H2A	0.354608	0.371724	0.008315	0.025*	
H2B	0.289017	0.496987	0.077765	0.025*	
C3	0.0821 (6)	0.3339 (4)	0.1059 (4)	0.0226 (8)	
H3A	0.010384	0.370283	0.175423	0.027*	
H3B	0.009678	0.363357	0.018959	0.027*	
C4	0.0989 (6)	0.1729 (4)	0.1496 (4)	0.0163 (7)	
H4A	0.121252	0.132901	0.069998	0.020*	
H4B	−0.022152	0.131202	0.196654	0.020*	
C5	0.9163 (6)	−0.2030 (4)	0.2504 (4)	0.0179 (7)	
H5A	0.902627	−0.175035	0.150356	0.022*	
H5B	1.036738	−0.159227	0.275978	0.022*	
C6	0.9258 (7)	−0.3639 (5)	0.3012 (5)	0.0325 (10)	
H6AA	1.003988	−0.405261	0.237893	0.039*	0.771 (13)
H6AB	0.988725	−0.390717	0.391775	0.039*	0.771 (13)
H6BC	0.911154	−0.407662	0.223452	0.039*	0.229 (13)
H6BD	1.055839	−0.392163	0.342976	0.039*	0.229 (13)
C7A	0.7197 (8)	−0.4183 (6)	0.3098 (6)	0.0265 (16)	0.771 (13)
H7AA	0.716201	−0.518002	0.364131	0.032*	0.771 (13)
H7AB	0.669637	−0.415264	0.217672	0.032*	0.771 (13)
C7B	0.7690 (16)	−0.4178 (16)	0.4028 (15)	0.016 (5)*	0.229 (13)
H7BA	0.732673	−0.515246	0.398840	0.019*	0.229 (13)
H7BB	0.819357	−0.422099	0.495122	0.019*	0.229 (13)
C8	0.5925 (6)	−0.3246 (4)	0.3767 (4)	0.0188 (8)	
H8AA	0.591371	−0.362462	0.476472	0.023*	0.771 (13)
H8AB	0.455767	−0.321214	0.343042	0.023*	0.771 (13)
H8BC	0.508814	−0.337978	0.458646	0.023*	0.229 (13)
H8BD	0.513653	−0.342444	0.300886	0.023*	0.229 (13)

### Atomic displacement parameters (Å^2^)

**Table:**

	*U*^11^	*U*^22^	*U*^33^	*U*^12^	*U*^13^	*U*^23^
Br1	0.01355 (17)	0.01735 (19)	0.01357 (17)	−0.00288 (13)	0.00077 (13)	−0.00496 (14)
Br2	0.01333 (17)	0.01052 (18)	0.02045 (19)	0.00292 (13)	−0.00208 (13)	−0.00219 (14)
Cu1	0.0167 (2)	0.0148 (2)	0.0137 (2)	0.00320 (17)	−0.00176 (16)	−0.00354 (17)
Cu2	0.0162 (2)	0.0132 (2)	0.0120 (2)	0.00180 (17)	−0.00123 (16)	−0.00298 (17)
S1	0.0142 (4)	0.0100 (4)	0.0108 (4)	0.0027 (3)	−0.0016 (3)	−0.0010 (3)
S2	0.0140 (4)	0.0111 (4)	0.0119 (4)	0.0016 (3)	−0.0012 (3)	−0.0037 (3)
C1	0.0254 (19)	0.0107 (18)	0.0198 (18)	−0.0041 (15)	−0.0019 (15)	−0.0003 (14)
C2	0.0212 (19)	0.0146 (19)	0.0218 (19)	0.0013 (15)	−0.0009 (15)	0.0036 (15)
C3	0.0189 (18)	0.018 (2)	0.027 (2)	0.0055 (15)	−0.0040 (16)	0.0000 (16)
C4	0.0198 (17)	0.0151 (18)	0.0138 (16)	0.0055 (14)	−0.0060 (14)	−0.0030 (14)
C5	0.0190 (18)	0.0183 (19)	0.0159 (17)	0.0070 (14)	0.0055 (14)	−0.0033 (14)
C6	0.038 (3)	0.021 (2)	0.041 (3)	0.0089 (19)	0.007 (2)	−0.012 (2)
C7A	0.036 (3)	0.018 (3)	0.028 (3)	−0.002 (2)	−0.010 (2)	−0.009 (2)
C8	0.0243 (19)	0.0141 (19)	0.0171 (17)	−0.0067 (15)	−0.0020 (15)	−0.0018 (14)

### Geometric parameters (Å, º)

**Table:**

Br1—Cu1	2.4755 (6)	C3—C4	1.520 (5)
Br1—Cu1^i^	2.5000 (6)	C4—H4A	0.9900
Br2—Cu2^ii^	2.5349 (6)	C4—H4B	0.9900
Br2—Cu2	2.4711 (6)	C5—H5A	0.9900
Cu1—Cu1^i^	3.3348 (10)	C5—H5B	0.9900
Cu1—S1	2.3292 (9)	C5—C6	1.522 (6)
Cu1—S2	2.2991 (10)	C6—H6AA	0.9900
Cu2—Cu2^ii^	2.9044 (9)	C6—H6AB	0.9900
Cu2—S1^iii^	2.2982 (9)	C6—H6BC	0.9900
Cu2—S2	2.2983 (9)	C6—H6BD	0.9900
S1—C1	1.837 (4)	C6—C7A	1.497 (7)
S1—C4	1.840 (4)	C6—C7B	1.488 (12)
S2—C5	1.831 (4)	C7A—H7AA	0.9900
S2—C8	1.833 (4)	C7A—H7AB	0.9900
C1—H1A	0.9900	C7A—C8	1.526 (6)
C1—H1B	0.9900	C7B—H7BA	0.9900
C1—C2	1.523 (5)	C7B—H7BB	0.9900
C2—H2A	0.9900	C7B—C8	1.484 (12)
C2—H2B	0.9900	C8—H8AA	0.9900
C2—C3	1.530 (5)	C8—H8AB	0.9900
C3—H3A	0.9900	C8—H8BC	0.9900
C3—H3B	0.9900	C8—H8BD	0.9900
			
Cu1—Br1—Cu1^i^	84.167 (19)	S1—C4—H4A	110.7
Cu2—Br2—Cu2^ii^	70.916 (19)	S1—C4—H4B	110.7
Br1—Cu1—Br1^i^	95.832 (19)	C3—C4—S1	105.3 (3)
S1—Cu1—Br1	107.82 (3)	C3—C4—H4A	110.7
S1—Cu1—Br1^i^	114.58 (3)	C3—C4—H4B	110.7
S2—Cu1—Br1	121.51 (3)	H4A—C4—H4B	108.8
S2—Cu1—Br1^i^	108.88 (3)	S2—C5—H5A	110.6
S2—Cu1—S1	108.12 (3)	S2—C5—H5B	110.6
Br2—Cu2—Br2^ii^	109.084 (19)	H5A—C5—H5B	108.7
Br2^ii^—Cu2—Cu2^ii^	53.516 (16)	C6—C5—S2	105.7 (3)
Br2—Cu2—Cu2^ii^	55.568 (17)	C6—C5—H5A	110.6
S1^iii^—Cu2—Br2	105.18 (3)	C6—C5—H5B	110.6
S1^iii^—Cu2—Br2^ii^	104.86 (3)	C5—C6—H6AA	110.2
S1^iii^—Cu2—Cu2^ii^	116.52 (3)	C5—C6—H6AB	110.2
S2—Cu2—Br2	104.11 (3)	C5—C6—H6BC	109.3
S2—Cu2—Br2^ii^	105.71 (3)	C5—C6—H6BD	109.3
S2—Cu2—Cu2^ii^	116.35 (3)	H6AA—C6—H6AB	108.5
S2—Cu2—S1^iii^	127.13 (4)	H6BC—C6—H6BD	108.0
Cu2^iii^—S1—Cu1	128.70 (4)	C7A—C6—C5	107.7 (4)
C1—S1—Cu1	108.21 (14)	C7A—C6—H6AA	110.2
C1—S1—Cu2^iii^	111.26 (13)	C7A—C6—H6AB	110.2
C1—S1—C4	94.46 (18)	C7B—C6—C5	111.5 (6)
C4—S1—Cu1	102.71 (12)	C7B—C6—H6BC	109.3
C4—S1—Cu2^iii^	105.46 (13)	C7B—C6—H6BD	109.3
Cu2—S2—Cu1	125.08 (4)	C6—C7A—H7AA	110.0
C5—S2—Cu1	107.52 (13)	C6—C7A—H7AB	110.0
C5—S2—Cu2	104.92 (13)	C6—C7A—C8	108.3 (4)
C5—S2—C8	93.98 (19)	H7AA—C7A—H7AB	108.4
C8—S2—Cu1	108.84 (13)	C8—C7A—H7AA	110.0
C8—S2—Cu2	111.74 (13)	C8—C7A—H7AB	110.0
S1—C1—H1A	110.6	C6—C7B—H7BA	109.4
S1—C1—H1B	110.6	C6—C7B—H7BB	109.4
H1A—C1—H1B	108.7	H7BA—C7B—H7BB	108.0
C2—C1—S1	105.9 (3)	C8—C7B—C6	111.0 (9)
C2—C1—H1A	110.6	C8—C7B—H7BA	109.4
C2—C1—H1B	110.6	C8—C7B—H7BB	109.4
C1—C2—H2A	110.5	S2—C8—H8AA	110.4
C1—C2—H2B	110.5	S2—C8—H8AB	110.4
C1—C2—C3	106.3 (3)	S2—C8—H8BC	111.3
H2A—C2—H2B	108.7	S2—C8—H8BD	111.3
C3—C2—H2A	110.5	C7A—C8—S2	106.8 (3)
C3—C2—H2B	110.5	C7A—C8—H8AA	110.4
C2—C3—H3A	110.3	C7A—C8—H8AB	110.4
C2—C3—H3B	110.3	C7B—C8—S2	102.3 (6)
H3A—C3—H3B	108.5	C7B—C8—H8BC	111.3
C4—C3—C2	107.3 (3)	C7B—C8—H8BD	111.3
C4—C3—H3A	110.3	H8AA—C8—H8AB	108.6
C4—C3—H3B	110.3	H8BC—C8—H8BD	109.2
			
Cu1—S1—C1—C2	91.5 (3)	S2—C5—C6—C7B	−3.4 (8)
Cu1—S1—C4—C3	−123.6 (2)	C1—S1—C4—C3	−13.8 (3)
Cu1—S2—C5—C6	−128.8 (3)	C1—C2—C3—C4	−49.3 (4)
Cu1—S2—C8—C7A	103.2 (3)	C2—C3—C4—S1	37.6 (4)
Cu1—S2—C8—C7B	143.3 (6)	C4—S1—C1—C2	−13.4 (3)
Cu2^iii^—S1—C1—C2	−121.9 (2)	C5—S2—C8—C7A	−6.8 (3)
Cu2^iii^—S1—C4—C3	99.7 (3)	C5—S2—C8—C7B	33.3 (6)
Cu2—S2—C5—C6	96.1 (3)	C5—C6—C7A—C8	−45.0 (5)
Cu2—S2—C8—C7A	−114.6 (3)	C5—C6—C7B—C8	30.0 (12)
Cu2—S2—C8—C7B	−74.5 (6)	C6—C7A—C8—S2	30.3 (5)
S1—C1—C2—C3	37.0 (4)	C6—C7B—C8—S2	−40.9 (11)
S2—C5—C6—C7A	38.3 (4)	C8—S2—C5—C6	−17.7 (3)

Symmetry codes: (i) −*x*+1, −*y*, −*z*; (ii) −*x*+2, −*y*, −*z*+1; (iii) −*x*+1, −*y*, −*z*+1.

### Hydrogen-bond geometry (Å, º)

**Table:**

*D*—H···*A*	*D*—H	H···*A*	*D*···*A*	*D*—H···*A*
C4—H4*B*···Br2^iv^	0.99	2.90	3.566 (4)	125
C5—H5*B*···Br2^ii^	0.99	2.89	3.556 (4)	126
C7*A*—H7*AA*···Br2^v^	0.99	2.95	3.885 (6)	157

Symmetry codes: (ii) −*x*+2, −*y*, −*z*+1; (iv) *x*−1, *y*, *z*; (v) *x*, *y*−1, *z*.
